# Effects of mobile health application interventions on glycolipid metabolism and quality of life in patients with type 2 diabetes mellitus: a systematic review and meta-analysis

**DOI:** 10.3389/fmed.2026.1894604

**Published:** 2026-07-29

**Authors:** Mingli Li, Shili Yang, Shuang Hu, Yanhui Chang, Xiangrong Liu

**Affiliations:** College of Nursing, Changchun University of Chinese Medicine, Changchun, China

**Keywords:** glycolipid metabolism, meta-analysis, mobile health applications, quality of life, type 2 diabetes mellitus

## Abstract

**Objective:**

This systematic review and meta-analysis of randomized controlled trials (RCTs) was conducted to comprehensively assess the impact of mobile health (mHealth) application interventions on glycolipid metabolism and quality of life among patients with type 2 diabetes mellitus (T2DM).

**Methods:**

A comprehensive search was conducted across major English- and Chinese-language electronic databases, including PubMed, Embase, the Cochrane Library, Web of Science, China National Knowledge Infrastructure (CNKI), Wanfang Data, VIP, and the Chinese Biomedical Literature Database, using a combination of MeSH terms and free words. The search period extended from the inception of each database to 12 March 2026. RCTs evaluating mHealth application interventions for T2DM were included. The risk of bias was assessed using the Cochrane risk of bias tool. Statistical analyses and heterogeneity tests were performed using Review Manager 5.4 and Stata 18.0. The primary outcome was glycated hemoglobin. The secondary outcomes included fasting blood glucose (FBG), 2-h postprandial blood glucose (2hPBG), total cholesterol (TC), triglycerides (TG), low-density lipoprotein cholesterol (LDL-C), high-density lipoprotein cholesterol (HDL-C), quality of life, and diabetes distress.

**Results:**

A total of 19 RCTs comprising 2,166 participants were included. The meta-analysis showed that, compared to control interventions, mHealth application-based management significantly reduced HbA1c [MD = −0.39, 95%CI: −0.49, −0.30], FBG [MD = −0.68, 95%CI: −0.89, −0.46], 2hPBG [MD = −1.46, 95%CI: −1.90, −1.02], and TC levels [MD = -0.21, 95%CI: −0.38, −0.05]. The intervention also improved quality of life [SMD = 0.24, 95%CI: 0.02, 0.47] and reduced diabetes distress [SMD = −0.22, 95%CI: −0.38, −0.05]. However, no statistically significant differences were observed between the groups regarding TG, HDL-C, or LDL-C levels.

**Conclusion:**

mHealth applications positively influence glucose metabolism and quality of life in patients with T2DM; however, their effects on lipid metabolism require further investigation.

**Systematic review registration:**

https://www.crd.york.ac.uk/PROSPERO/view/CRD420261354082, Unique Identifier: CRD420261354082.

## 1 Introduction

Diabetes mellitus is a chronic metabolic disorder characterized by insufficient insulin secretion or insulin deficiency, leading to hyperglycemia and disturbances in glucose, lipid, and protein metabolism (1). In this study, ‘glycolipid metabolism’ refers to the integrated metabolic pathways of glucose and lipids, operationalized through glycemic indicators [e.g., HbA1c, fasting blood glucose (FBG), and 2-h postprandial blood glucose (2hPBG)], along with lipid parameters [total cholesterol (TC), triglycerides (TG), low-density lipoprotein cholesterol (LDL-C), and high-density lipoprotein cholesterol (HDL-C)]. These markers are critical for assessing metabolic control and cardiovascular risk in patients with type 2 diabetes mellitus (T2DM). According to the 11th edition of the Global Diabetes Atlas published by the International Diabetes Federation (IDF) in 2025, there were 589 million adults with diabetes worldwide in 2024, a figure projected to reach 853 million by 2050. China has approximately 148 million diabetes patients, the highest in the world, with type 2 diabetes mellitus (T2DM) accounting for more than 90% of cases (2). As the disease progresses, it can lead to severe multi-organ complications, reduce patients’ quality of life, and impose a heavy burden on healthcare systems and societies, making it a major public health challenge (3).

Effective management of T2DM is essential for achieving glycemic control and relies on lifestyle interventions, pharmacotherapy, and continuous patient education to control the disease and prevent complications (4). However, current diabetes management faces challenges, such as poor patient self-management awareness, a lack of knowledge regarding blood glucose monitoring and healthy lifestyle practices, geographical and temporal constraints, insufficient supervision and timely feedback, and inadequate diabetes education coverage. These barriers hinder the implementation of comprehensive diabetes management and long-term disease control.

With the advancement of digital health technologies, mobile health (mHealth) applications offer advantages such as convenience, flexibility, personalization, and broad accessibility. These can provide continuous glucose monitoring, health education, dietary and exercise guidance, medication reminders, and other intelligent services, better meeting the personalized long-term management needs of patients while reducing traditional geographical and time-related barriers (5).

Although several systematic reviews have examined the effects of remote interventions on HbA1c levels, self-management behaviors, and quality of life in patients with diabetes (6–8), there remains insufficient evidence regarding the specific effects of mHealth applications on glycolipid metabolism and quality of life in patients with T2DM. Moreover, the majority of previous reviews included studies involving complex or heterogeneous intervention technologies, often combining smartphone applications with other technologies, such as text messaging or wearable devices, which makes it difficult to isolate the standalone effects of mHealth applications. Given the unique advantages of mHealth applications in terms of efficiency, interactivity, and personalized management, it is essential to evaluate their independent effects. Furthermore, no prior meta-analysis has systematically synthesized evidence on comprehensive lipid profile outcomes (TC, TG, LDL-C, and HDL-C) alongside glycemic indicators and patient-reported outcomes, including both quality of life and diabetes distress. Therefore, this systematic review aimed to comprehensively evaluate the effects of mHealth applications on glycolipid metabolism and quality of life among patients with T2DM, providing evidence for optimizing digital health management in this population.

## 2 Methods

This systematic review adhered to the Preferred Reporting Items for Systematic Reviews and Meta-Analyses (PRISMA) guidelines (9), and its protocol was registered with PROSPERO (CRD420261354082).

### 2.1 Search strategy

A systematic search was performed across the following databases from their inception to 12 March 2026: PubMed, Web of Science, the Cochrane Library, Embase, China National Knowledge Infrastructure (CNKI), Wanfang Data, VIP, and the Chinese Biomedical Literature Database. The search strategy employed a combination of MeSH terms and free words: (“diabetes mellitus, type 2” OR “diabetes mellitus, noninsulin-dependent” OR “type 2 diabetes” OR “diabetes, type 2”) AND (“mobile applications” OR “application, mobile” OR “mobile apps” OR “portable software apps” OR “app”) AND (“randomized controlled trial” OR “randomized” OR “placebo”). The PubMed search strategy is presented as an example in Table 1.

**Table 1:** Search strategy for PubMed.

Search	Query
#1	“Diabetes Mellitus, Type 2”[MeSH Terms]
#2	“Diabetes Mellitus, Noninsulin-Dependent”[Title/Abstract] OR “Diabetes Mellitus, Ketosis-Resistant”[Title/Abstract] OR “Diabetes Mellitus, Ketosis Resistant”[Title/Abstract] OR “Ketosis-Resistant Diabetes Mellitus”[Title/Abstract] OR “Diabetes Mellitus, Non Insulin Dependent”[Title/Abstract] OR “Diabetes Mellitus, Non-Insulin-Dependent”[Title/Abstract] OR “Non-Insulin-Dependent Diabetes Mellitus”[Title/Abstract]OR “Diabetes Mellitus, Stable”[Title/Abstract] OR “Stable Diabetes Mellitus”[Title/Abstract] OR “Diabetes Mellitus, Type II”[Title/Abstract] OR “NIDDMDiabetes Mellitus, Noninsulin Dependent”[Title/Abstract] OR “Diabetes Mellitus, Maturity-Onset”[Title/Abstract] OR “Diabetes Mellitus, Maturity Onset”[Title/Abstract]OR “Maturity-Onset Diabetes Mellitus”[Title/Abstract] OR “Maturity Onset Diabetes Mellitus”[Title/Abstract] OR “MODY”[Title/Abstract] OR “Diabetes Mellitus, Slow-Onset”[Title/Abstract] OR “Diabetes Mellitus, Slow Onset”[Title/Abstract] OR “Slow-Onset Diabetes Mellitus”[Title/Abstract]OR “Type 2 Diabetes Mellitus”[Title/Abstract] OR “Noninsulin-Dependent Diabetes Mellitus”[Title/Abstract] OR “Noninsulin Dependent Diabetes Mellitus”[Title/Abstract] OR “Maturity-Onset Diabetes”[Title/Abstract] OR “Diabetes, Maturity-Onset”[Title/Abstract] OR “Maturity Onset Diabetes”[Title/Abstract]OR “Type 2 Diabetes”[Title/Abstract] OR “Diabetes, Type 2”[Title/Abstract] OR “Diabetes Mellitus, Adult-Onset”[Title/Abstract] OR “Adult-Onset Diabetes Mellitus”[Title/Abstract]OR “Diabetes Mellitus, Adult Onset”[Title/Abstract]
#3	#1 OR #2
#4	“Mobile Applications”[MeSH Terms]
#5	“Application, Mobile”[Title/Abstract] OR “Applications, Mobile”[Title/Abstract] OR “Mobile Application”[Title/Abstract] OR “Mobile Apps”[Title/Abstract] OR “App, Mobile”[Title/Abstract] OR “Apps, Mobile”[Title/Abstract] OR “Mobile App”[Title/Abstract] OR “Portable Software Apps”[Title/Abstract] OR “App, Portable Software “[Title/Abstract] OR “Portable Software App”[Title/Abstract]OR “Software App, Portable”[Title/Abstract] OR “Portable Software Applications”[Title/Abstract] OR “Application, Portable Software”[Title/Abstract] OR “Portable Software Application”[Title/Abstract] OR “Software Application, Portable”[Title/Abstract] OR “Smartphone Apps”[Title/Abstract] OR “App, Smartphone”[Title/Abstract] OR “Apps, Smartphone”[Title/Abstract] OR “Smartphone App”[Title/Abstract]OR “Portable Electronic Apps”[Title/Abstract] OR “App, Portable Electronic”[Title/Abstract] OR “Electronic App, Portable”[Title/Abstract] OR “Portable Electronic App”[Title/Abstract] OR “Portable Electronic Applications”[Title/Abstract] OR “Application, Portable Electronic”[Title/Abstract] OR “Electronic Application, Portable”[Title/Abstract] OR “Portable Electronic Application “[Title/Abstract]
#6	#4 OR #5
#7	“randomized controlled trial”[Publication Type]
#8	“randomized”[Title/Abstract] OR “placebo”[Title/Abstract]
#9	#7 OR #8
#10	#3 AND #6 AND #9

### 2.2 Inclusion and exclusion criteria

#### 2.2.1 Inclusion criteria

The inclusion criteria were as follows:

(1) Participants: Adults aged ≥18 years with a confirmed diagnosis of T2DM and no mental illness.
(2) Intervention: Studies in which the intervention group received an mHealth application intervention.
(3) Comparator: Studies in which the control group received usual care without any mobile health application intervention.
(4) Outcomes: Studies reporting HbA1c as the primary outcome and at least one secondary outcome (FBG, 2hPBG, TC, TG, LDL-C, HDL-C, quality of life, or diabetes distress).
(5) Study design: Randomized controlled trials (RCTs).

#### 2.2.2 Exclusion criteria

The exclusion criteria were as follows:

(1) Studies involving participants with mixed diabetes types (e.g., type 1 diabetes, gestational diabetes, or prediabetes).
(2) Studies in which the intervention was not based on mHealth applications, such as text messages, phone calls, or social media applications (e.g., WeChat or QQ).
(3) Studies involving the simultaneous use of multiple applications.
(4) Studies in which the intervention included additional components (e. g., text messaging or wearable devices).

### 2.3 Study selection and data extraction

Any disagreements were resolved by consulting a third reviewer. Following removal of duplicates using NoteExpress software, titles and abstracts were screened, and the full texts of potentially eligible studies were subsequently assessed. The extracted information encompassed the first author, publication year, country, ethnicity, sample size, participant characteristics, intervention details, control group measures, intervention duration, and outcomes.

### 2.4 Quality assessment

In total, two reviewers assessed the methodological quality of the included RCTs using the Cochrane risk-of-bias tool (10), which covers seven domains: Random sequence generation, allocation concealment, blinding of participants and personnel, blinding of outcome assessment, incomplete outcome data, selective outcome reporting, and other sources of bias. Disagreements were resolved by consensus through discussion with a third reviewer. Each domain was rated as low risk, unclear risk, or high risk.

### 2.5 GRADE evidence assessment

This study employed the GRADE approach to assess the certainty of evidence for the following outcomes: HbA1c, FBG, 2hPBG, TC, TG, LDL-C, HDL-C, quality of life, and diabetes distress. The certainty of evidence was graded into four levels—high, moderate, low, and very low—based on the following five domains: Study limitations, inconsistency, indirectness, imprecision, and publication bias (11). Furthermore, two researchers independently performed the quality appraisal; discrepancies were first resolved through discussion and consultation, and if no consensus could be reached, a third reviewer made the final decision.

### 2.6 Statistical analysis

Data were analyzed using Review Manager 5.4 and Stata 18.0. Continuous outcomes were reported as mean ± standard deviation (SD). When the SD was not directly reported, we calculated it from available data (e.g., standard errors, confidence intervals, or interquartile ranges) using established formulas (12). Studies that did not report sufficient data for mean and SD calculation and did not respond to our data request were excluded from the relevant meta-analyses. For outcomes measured using the same scale, mean differences (MDs) were used as effect sizes; for those measured with different instruments, standardized mean differences (SMDs) were used. All effect sizes were reported with 95% confidence intervals (CIs) (13). Statistical heterogeneity was assessed using I^2^ and the chi-squared test. The choice between fixed effects and random effects models was based on both statistical heterogeneity and clinical considerations. A fixed effects model (inverse-variance method) was used when heterogeneity was low (I^2^ ≤ 50% and *p* > 0.10), under the assumption that studies share a common true effect. A random effects model (DerSimonian–Laird method) was used when moderate or substantial heterogeneity was present (I^2^ > 50% or *p* ≤ 0.10), as this model incorporates between-study variance and is more appropriate when studies differ in intervention characteristics, populations, or other factors (14). Sensitivity analyses were performed to assess the robustness of the results by sequentially removing each study, and publication bias was evaluated using Egger’s test (15).

## 3 Results

### 3.1 Literature search results

**Figure 1:**
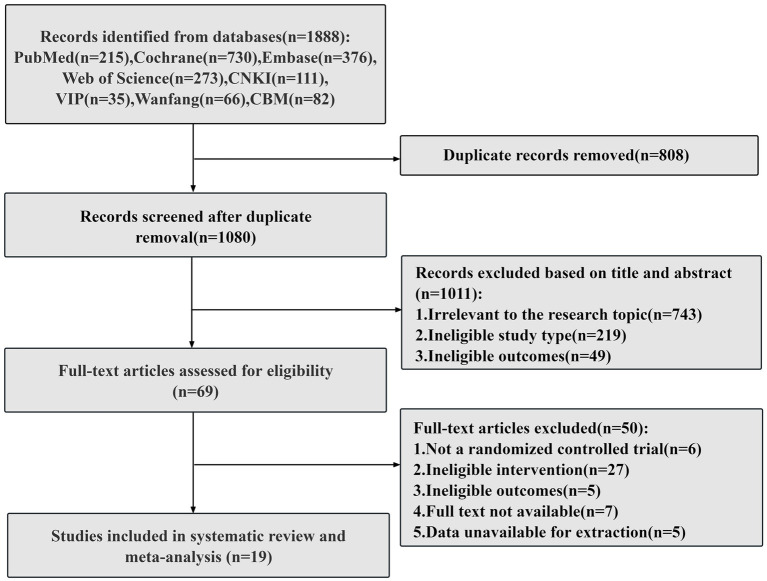
Flow diagram of literature screening.

A total of 1,888 records were identified from the database searches: PubMed (215), the Cochrane Library (730), Embase (376), Web of Science (273), CNKI (111), VIP (35), Wanfang Data (66), and the Chinese Biomedical Literature Database (82). After removing 808 duplicates, 1,080 records remained. Title and abstract screening excluded 1,011 records, leaving 69 full-text articles for assessment. Ultimately, 19 studies met the inclusion criteria. The literature screening flow diagram is shown in Figure 1.

### 3.2 Characteristics of included studies

The 19 RCTs were published between 2014 and 2025, comprising 2,166 participants. Intervention durations ranged from 3 to 12 months. All interventions were mHealth application-based health management programs that incorporated various self-management support components, including health recording, education, and behavioral feedback, while control groups received usual care. As shown in Table 2, participants were middle-aged (45–61 years). Geographically, 11 studies (57.89%) were conducted in Asia (16–26), five (26.32%) in Europe (4, 27–30), two (10.53%) in North America (31, 32), and one (5.26%) in Oceania (33). The predominant ethnicities were Asian (11 studies, 57.9%) (16–26), followed by white (six studies, 31.6%) (4, 27–30, 33), with two studies (10.5%) with mixed ethnicity populations (31, 32).

**Table 2:** Characteristics of included studies.

Author/year	Country	Ethnicity	Age (mean±SD, intervention/control)	Sample size (I/C)	Intervention	mHealth app intervention content	Control measures	Intervention duration	Outcomes
Wang et al., 2019 (16)	China	Asian	45.13 ± 7.83/45.80 ± 8.38	60/60	Usual care + mobile app	① Health recording and personalized feedback; ② Health education; ③ Patient–provider communication; ④ Follow-up management; ⑤ Patient community; ⑥ Anomaly alerts	Routine discharge guidance and follow-up	6 months	①②③
Bretschneider et al., 2025 (4)	Germany	White	61.0 ± 10.7/60.2 ± 10.9	73/76	Usual diabetes care+Vitadio APP	① Self-management education; ② Daily motivational messages; ③ Peer support; ④ Personalized goal setting; ⑤ Health monitoring	Usual care	6 months	①⑨
Enyin et al., 2020 (33)	Australia	White	55.4 ± 9.7/58.4 ± 10.5	79/83	Usual care+My Diabetes Coach APP	① Health recording; ② Health monitoring and personalized guidance; ③ Professional consultation; ④ Peer interaction	Usual care	12 months	①⑧⑨
Lee et al., 2022 (17)	South Korea	Asian	51.3 ± 13.1/52.6 ± 12.1	91/87	Usual care+iCareD APP	① Blood glucose monitoring; ② Personalized guidance; ③ Health education	Usual care	22 weeks	①②⑦
Höchsmann et al., 2019 (27)	Switzerland	White	57.0 ± 5.2/59.0 ± 6.7	18/17	Usual care+Mission: Schweinehund APP	① Mobile games; ② Narrative motivation; ③ Personalized exercise; ④ Behavioral techniques	Usual care	6 months	①④⑤⑥⑦
Quinn et al., 2011 (31)	USA	Mixed	53.2 ± 8.4/53.7 ± 8.2	23/56	Usual care + mobile app	① Health recording; ② Personalized education and behavioral reminders	Usual care	12 months	①④⑤⑥⑦⑨
Yang et al., 2020 (18)	South Korea	Asian	60.6 ± 10.2/54.1 ± 10.1	145/94	Usual outpatient care + smartphone-based glucose monitoring and feedback system	① Health recording; ② Blood glucose monitoring; ③ Personalized diabetes management goals; ④ Personalized guidance	Usual outpatient care	3 months	①②④⑤⑥⑦
Holmen et al., 2014 (28)	Norway	White	55.9 ± 12.2/58.6 ± 11.8	39/41	Usual care +Few Touch Application	① Health recording; ② Personal goal management; ③ Health information	Usual care	12 months	①
Hilmarsdóttir et al., 2021 (29)	Iceland	White	50.9 ± 11.8/51.5 ± 9.5	15/15	Usual standard care+SidekickHealth APP	① Health recording; ② Stress management; ③ Personalized support; ④ Game-based motivation	Usual care	6 months	①④⑤⑥⑦⑧⑨
Wongdama et al., 2025 (19)	Thailand	Asian	54.6 ± 14.3/61.9 ± 11.9	60/63	Usual care+Rama Diabetes Care APP	① Health monitoring; ② Medication reminders; ③ Physical activity tracking; ④ Symptom screening; ⑤ Dietary recording	Self-management education and support	6 months	①②⑦⑧
Agarwal et al., 2019 (32)	Canada	Mixed	51.5 ± 10.6/52.1 ± 10.7	69/77	Usual care+BlueStar APP	① Health recording; ② Personalized health information delivery; ③ Clinical report generation	Usual care	6 months	①⑧⑨
Kleinman et al., 2017 (20)	India	Asian	48.8 ± 9.0/48.0 ± 9.5	44/47	Usual care+Gather mHealth APP	① Medication management; ② Blood glucose monitoring; ③ Personalized information delivery; ④ Patient–provider communication; ⑤ Smart alerts	Usual care	6 months	①②
Kusnanto et al., 2019 (21)	Indonesia	Asian	52.6 ± 6.8/50.5 ± 5.9	15/15	Usual diabetes self-management education+ DM-calendar APP	① Blood glucose monitoring reminders; ② Health education content delivery	Usual care	3 months	①②④⑤⑥⑦
Zhai et al., 2020 (22)	China	Asian	54.12 ± 11.1/55.64 ± 14.2	60/58	Usual care+ “Yi Tang Yi Hu”app	① Blood glucose monitoring; ② Medication/dietary guidance; ③ Emotional management; ④ Patient–provider communication; ⑤ Hyperglycemia/hypoglycemia alerts	Usual care	6 months	①
Ruiz-Leon et al., 2025 (30)	Spain	White	56.0 ± 10.8/60.5 ± 8.11	50/53	Usual care+ Greenhabit APP	① Health challenge and information delivery; ② Social support; ③ Personalized dietary advice; ④ Follow-up management	Healthy dietary advice + usual care	3 months	①②④⑤⑥⑦⑨
Zhang, 2018 (23)	China	Asian	58.6 ± 8.3/57.9 ± 7.8	68/60	Usual care+ “Da Tang Yi”app	① Health education; ② Health recording; ③ Blood glucose monitoring; ④ Remote guidance by healthcare providers; ⑤ Personalized guidance	Usual care	6 months	①②③
Shu et al., 2020 (24)	China	Asian	45 ± 9/47 ± 10	58/57	Usual care+“Yi Yun Jian Kang”app	① Health records; ② Anomaly data alerts; ③ Online patient–provider and patient–patient communication; ④ Diabetes health education content delivery	Usual care	6 months	①②③⑦
Liu et al., 2022 (25)	China	Asian	54 ± 13/55 ± 12	60/60	Usual care + mobile app	① Health recording and personalized feedback; ② Family member binding; ③ Points-based incentives; ④ Health education; ⑤ Patient–provider interaction	Usual care	6 months	①②③
Hu et al., 2018 (26)	China	Asian	52.3 ± 6.8/53.1 ± 7.2	60/60	Usual care+“Mai Ya Tang”app	① Health knowledge; ② Anomaly reporting; ③ Personalized plan formulation; ④ Medication reminders; ⑤ Medication information query	Usual care	6 months	①②④⑤

Outcomes: ① HbA1c;② FBG;③ 2hPBG;④ TC;⑤ TG;⑥ HDL-C; ⑦ LDL-C;⑧ Quality of life;⑨ Diabetes distress.

### 3.3 Risk of bias assessment

**Figure 2:**
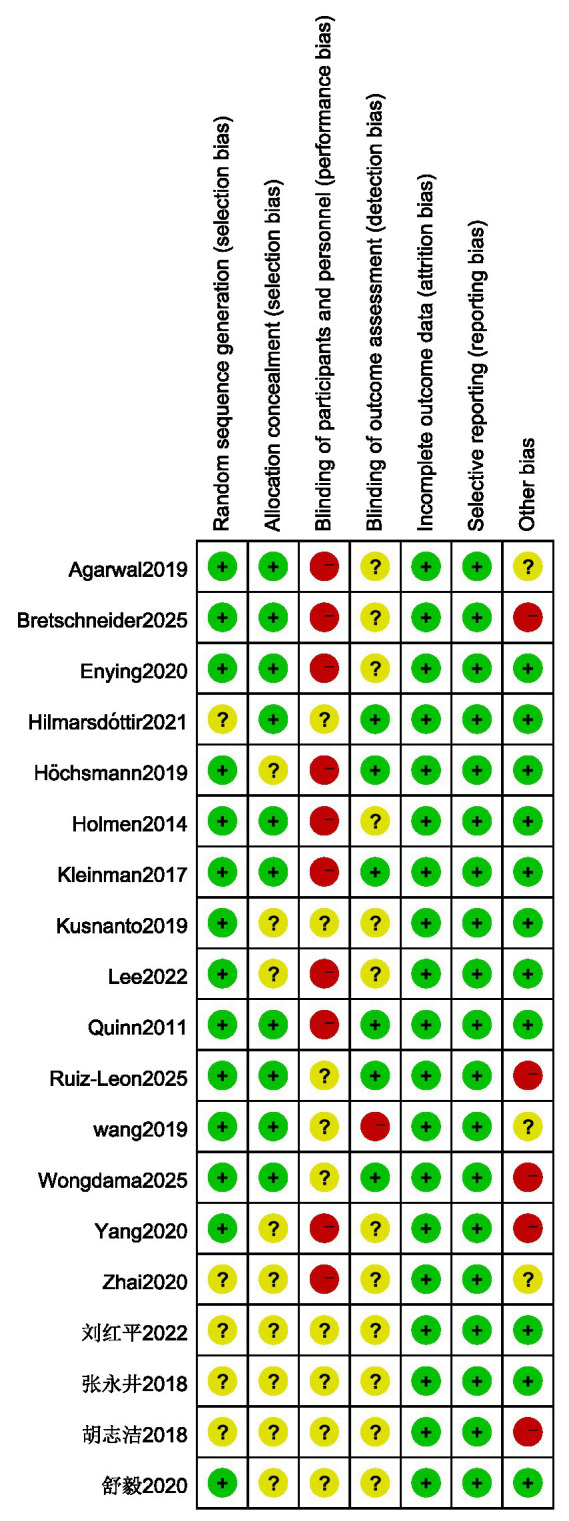
Risk of bias assessment of included studies.

**Figure 3:**
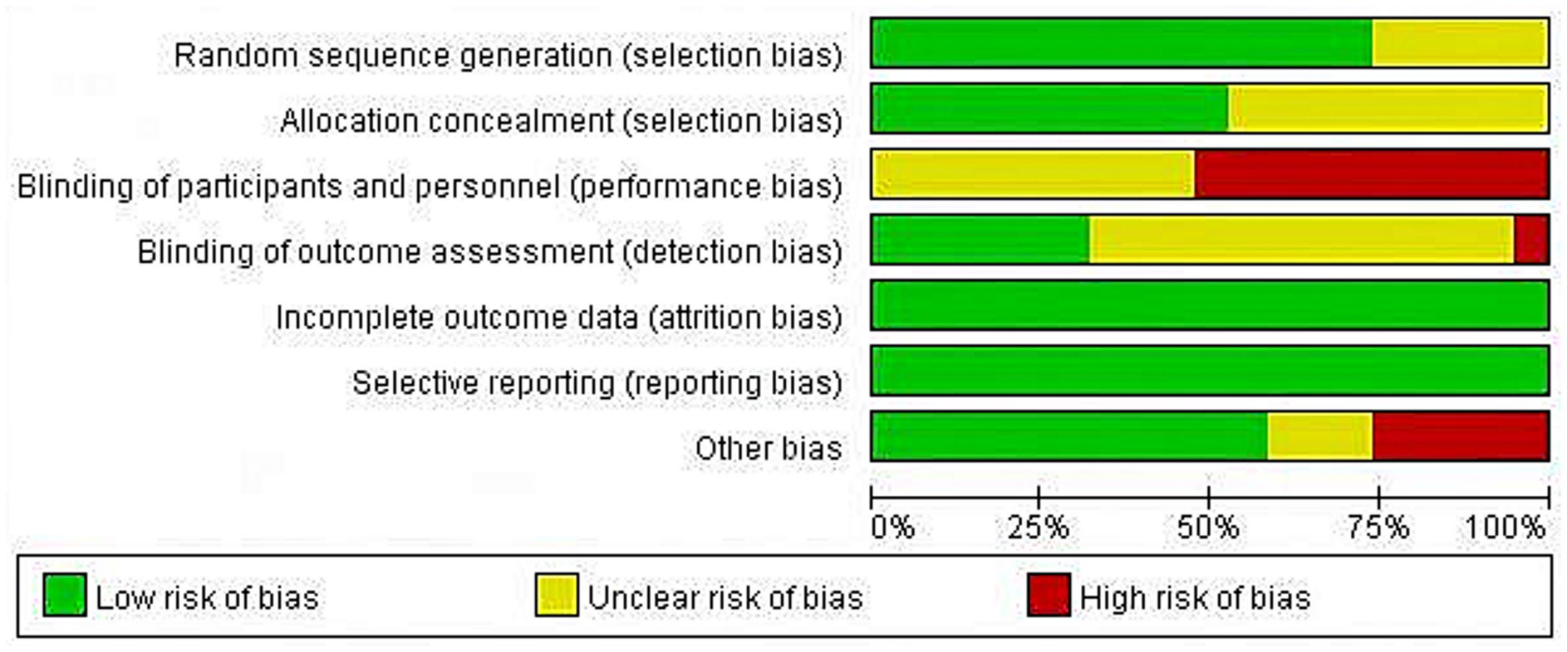
Proportion of risk of bias.

Among the 19 RCTs, 14 (73.7%) adequately reported random sequence generation, and 10 RCTs (52.6%) described allocation concealment. Due to the digital nature of mHealth interventions, blinding of participants and personnel was challenging: 10 studies were rated as having a high risk of bias, while nine studies were rated as having an unclear risk of bias in this domain. For blinding of outcome assessors, six studies were rated as having a low risk of bias, one as having a high risk of bias, and 12 as having an unclear risk of bias. All studies were rated as having a low risk of bias for selective outcome reporting and incomplete outcome data. For other sources of bias, 11 studies were rated as having a low risk of bias, five as having a high risk of bias, and three as having an unclear risk of bias. Figures 2, 3 present the risk of bias summary and proportions.

### 3.4 Functions of mHealth applications

The functions of the mHealth applications were categorized as follows: Health recording and monitoring, health education and personalized guidance, communication with healthcare providers and follow-up management, behavioral incentives and reminders, and medication and self-management support. Furthermore, two main modes of interaction were identified: (1) automatic feedback and reminders based on prespecified algorithms using patient-uploaded data (e.g., blood glucose, diet, and physical activity) and (2) remote personalized guidance by healthcare providers via back-end access to patient data. All 19 studies (100%) provided health recording and monitoring functions; 17 (89.5%) included health education or personalized guidance, 12 (63.2%) incorporated communication or follow-up modules, and 10 (52.6%) used incentives or reminders to support behavioral management. The aforementioned functions, including health monitoring, personalized guidance, and interactive communication, serve as the core vehicles through which mobile health applications enhance effective self-management capabilities in patients with type 2 diabetes mellitus (T2DM). By providing continuous supervision and real-time feedback, these applications regulate patients’ behaviors regarding diet, physical activity, blood glucose monitoring, and medication adherence, thereby contributing to improved glycemic and lipid metabolic control and enhanced quality of life.

### 3.5 Effect of mHealth applications on HbA1c

**Figure 4:**
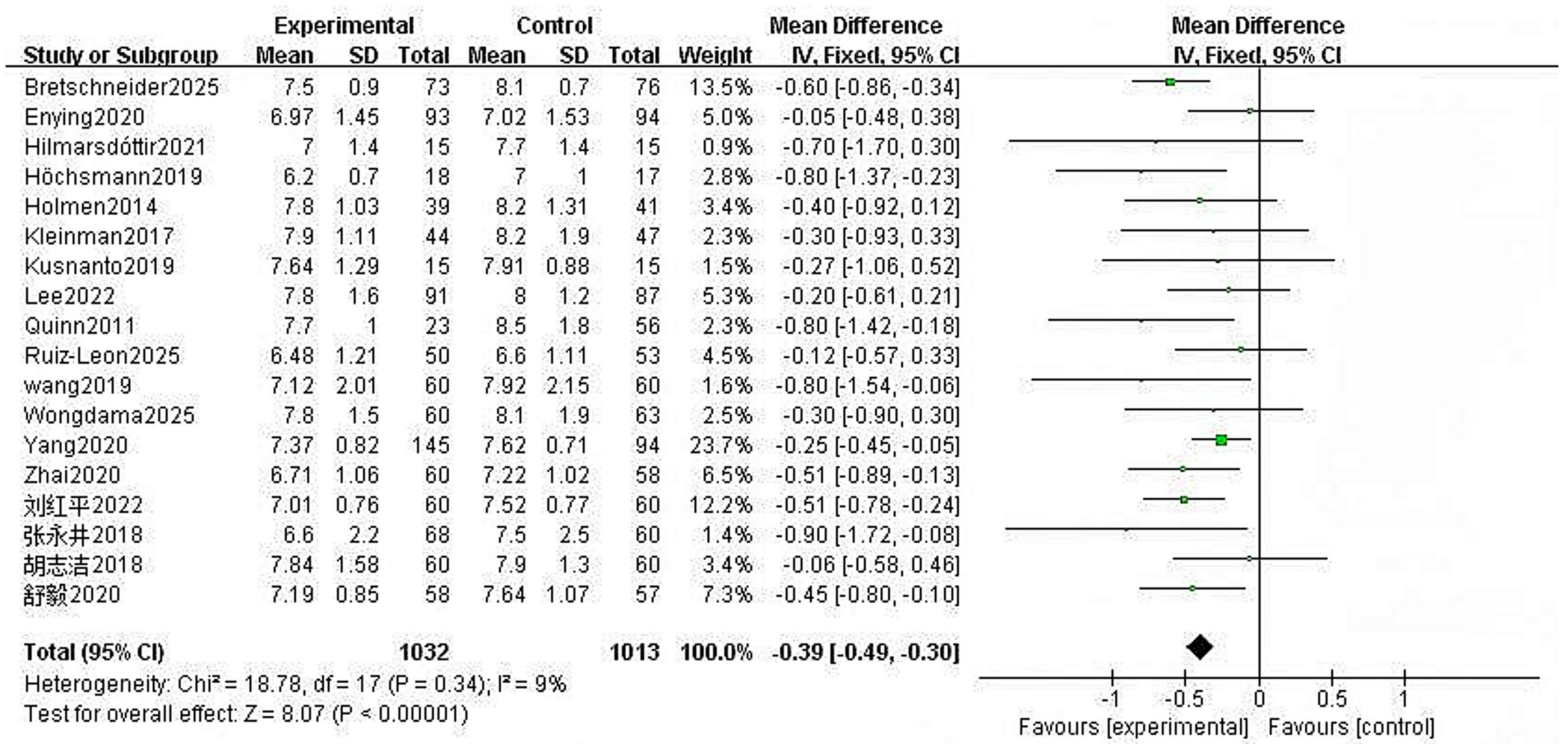
Effect of mobile health applications on HbA1c.

The overall meta-analysis showed low heterogeneity among the studies (I^2^ = 9%, *p* = 0.34), so a fixed effects model was used. Compared to controls, mHealth application interventions significantly reduced HbA1c levels [MD = -0.39, 95%CI (−0.49, −0.30), *p* < 0.00001] (Figure 4).

### 3.6 Effects on secondary outcomes

#### 3.6.1 Fasting blood glucose

**Figure 5:**
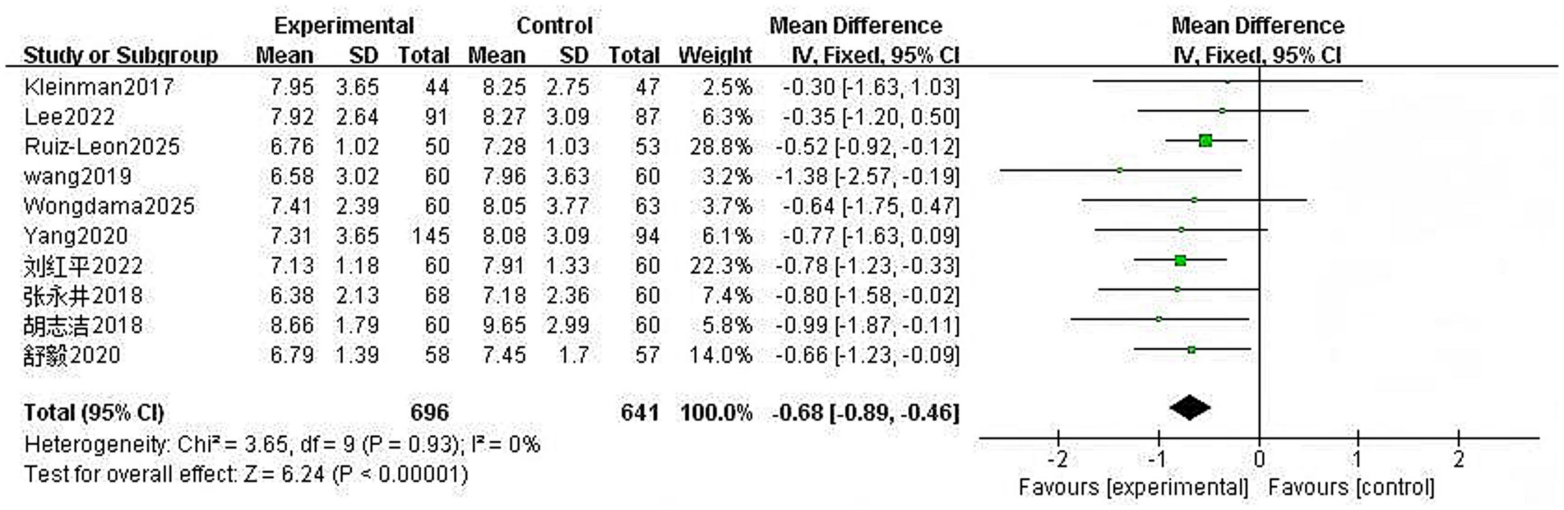
Effect of mobile health applications on FBG.

A total of 10 studies (16–20, 23–26, 30) reported FBG levels. No heterogeneity was observed (I^2^ = 0%, *p* = 0.93); therefore, a fixed effects model was used. The intervention group had significantly lower FBG levels than the control group [MD = −0.68, 95%CI (−0.89, −0.46), *p* < 0.00001] (Figure 5).

#### 3.6.2 2-hour postprandial blood glucose

**Figure 6:**
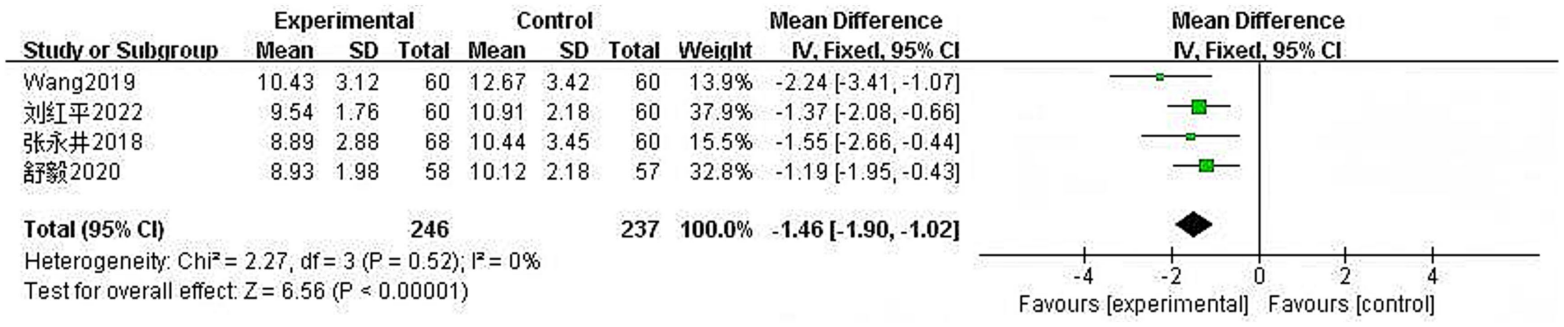
Effect of mobile health applications on 2hPBG.

In total, four studies (16, 23–25) reported 2hPBG levels. No heterogeneity was observed (I^2^ = 0%, *p* = 0.52); therefore, a fixed effects model was used. The intervention group had significantly lower 2hPBG levels than the control group [MD = −1.46, 95%CI (−1.90, −1.02), *p* < 0.00001] (Figure 6).

#### 3.6.3 Total cholesterol

**Figure 7:**
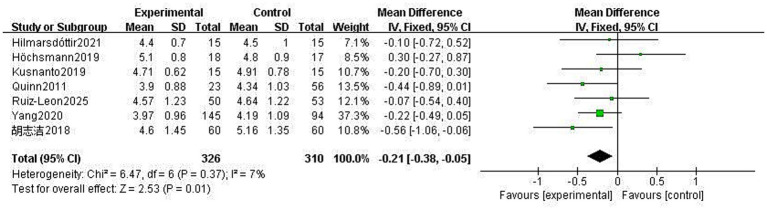
Effect of mobile health applications on TC.

A total of seven studies (18, 21, 26, 27, 29–31) reported TC levels. Heterogeneity was low (I^2^ = 7%, *p* = 0.37); therefore, a fixed effects model was used. The intervention group had significantly lower TC levels than the control group [MD = -0.21, 95%CI (−0.38, −0.05), *p* = 0.01] (Figure 7).

#### 3.6.4 Triglycerides

**Figure 8:**
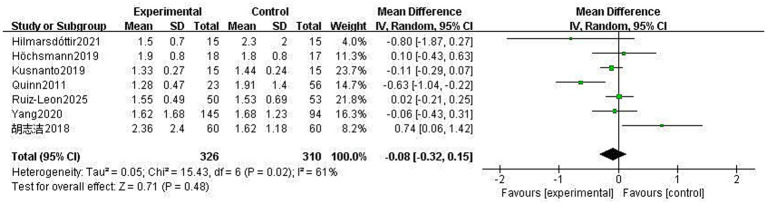
Effect of mobile health applications on TG.

A total of seven studies (18, 21, 26, 27, 29–31) reported TG levels. Heterogeneity was substantial (I^2^ = 61%, *p* = 0.02); therefore, a random effects model was used. No significant difference was found between the groups [MD = -0.08, 95%CI (−0.32, 0.15), *p* = 0.48] (Figure 8). A sensitivity analysis performed by sequentially removing one study at a time showed that after excluding the study by Quinn et al., heterogeneity decreased (I^2^ = 38%, *p* = 0.15).

#### 3.6.5 High-density lipoprotein cholesterol

**Figure 9:**
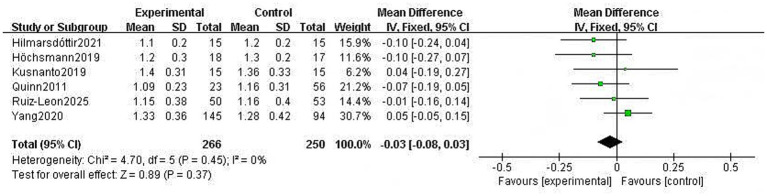
Effect of Mobile Health Applications on HDL-C.

In total, six studies (18, 21, 27, 29–31) reported HDL-C levels. No heterogeneity was observed (I^2^ = 0%, *p* = 0.45); therefore, a fixed effects model was used. No significant difference was found between the groups [MD = -0.03, 95%CI (−0.08, 0.03), *p* = 0.37] (Figure 9).

#### 3.6.6 Low-density lipoprotein cholesterol

**Figure 10:**
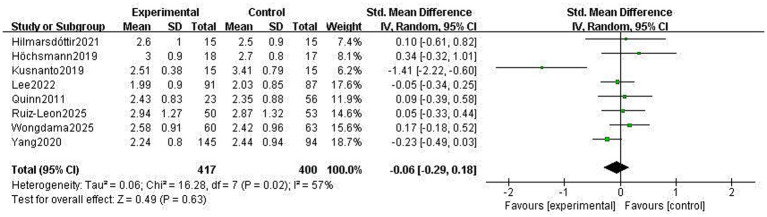
Effect of mobile health applications on LDL-C.

In total, eight studies (17–19, 21, 27, 29–31) reported LDL-C levels. Heterogeneity was substantial (I^2^ = 57%, *p* = 0.02); therefore, a random effects model was used. No significant difference was found between the groups [MD = -0.06, 95%CI (−0.29, 0.18), *p* = 0.63] (Figure 10). A sensitivity analysis showed that after excluding the study by Kusnanto et al., heterogeneity decreased (I^2^ = 0%, *p* = 0.51).

#### 3.6.7 Quality of life

**Figure 11:**
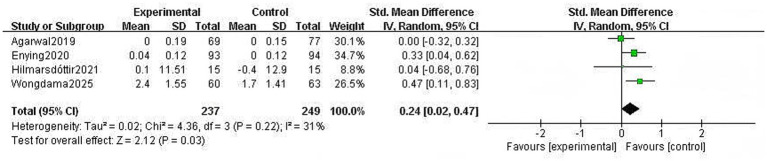
Effect of mobile health applications on quality of life.

A total of four studies (19, 29, 32, 33) reported quality of life using different instruments (EQ-5D, AQoL-8D, and the IQL test). The use of SMD as the effect size measure is statistically appropriate because the SMD standardizes effect sizes across different measurement scales by expressing the mean difference in units of the pooled standard deviation, allowing for a meaningful synthesis of outcomes measured using different instruments. The low heterogeneity (I^2^ = 31%, *p* = 0.22) further supports the combinability of these studies, indicating that despite using different instruments, the studies produced reasonably consistent effect estimates. The meta-analysis showed that mHealth application interventions significantly improved quality of life [SMD = 0.24, 95%CI(0.02, 0.47), *p* = 0.03] (Figure 11). A sensitivity analysis excluding the study by Agarwal et al. (27) reported reduced heterogeneity (I^2^ = 0%, *p* = 0.56), indicating the robustness of the results.

#### 3.6.8 Diabetes distress

**Figure 12:**
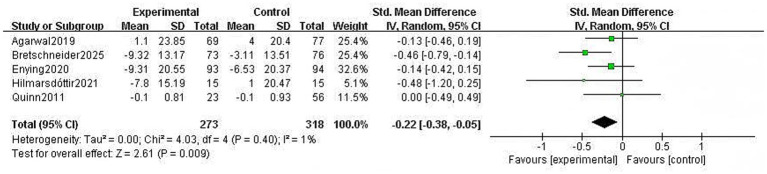
Effect of mobile health applications on diabetes distress.

A total of five studies (4, 29, 31–33) reported diabetes distress using either the Problem Areas in Diabetes (PAID) scale or the Diabetes Distress Scale (DDS). Heterogeneity was low (I^2^ = 1%, *p* = 0.40); therefore, a fixed effects model with SMD was used. The intervention group had significantly lower diabetes distress than the control group [SMD = -0.22, 95%CI(−0.38, −0.05), *p* = 0.009] (Figure 12).

### 3.7 Publication bias

Egger’s test for HbA1c (*p* = 0.205) indicated no significant publication bias.

### 3.8 GRADE evidence assessment

**Figure 13:**
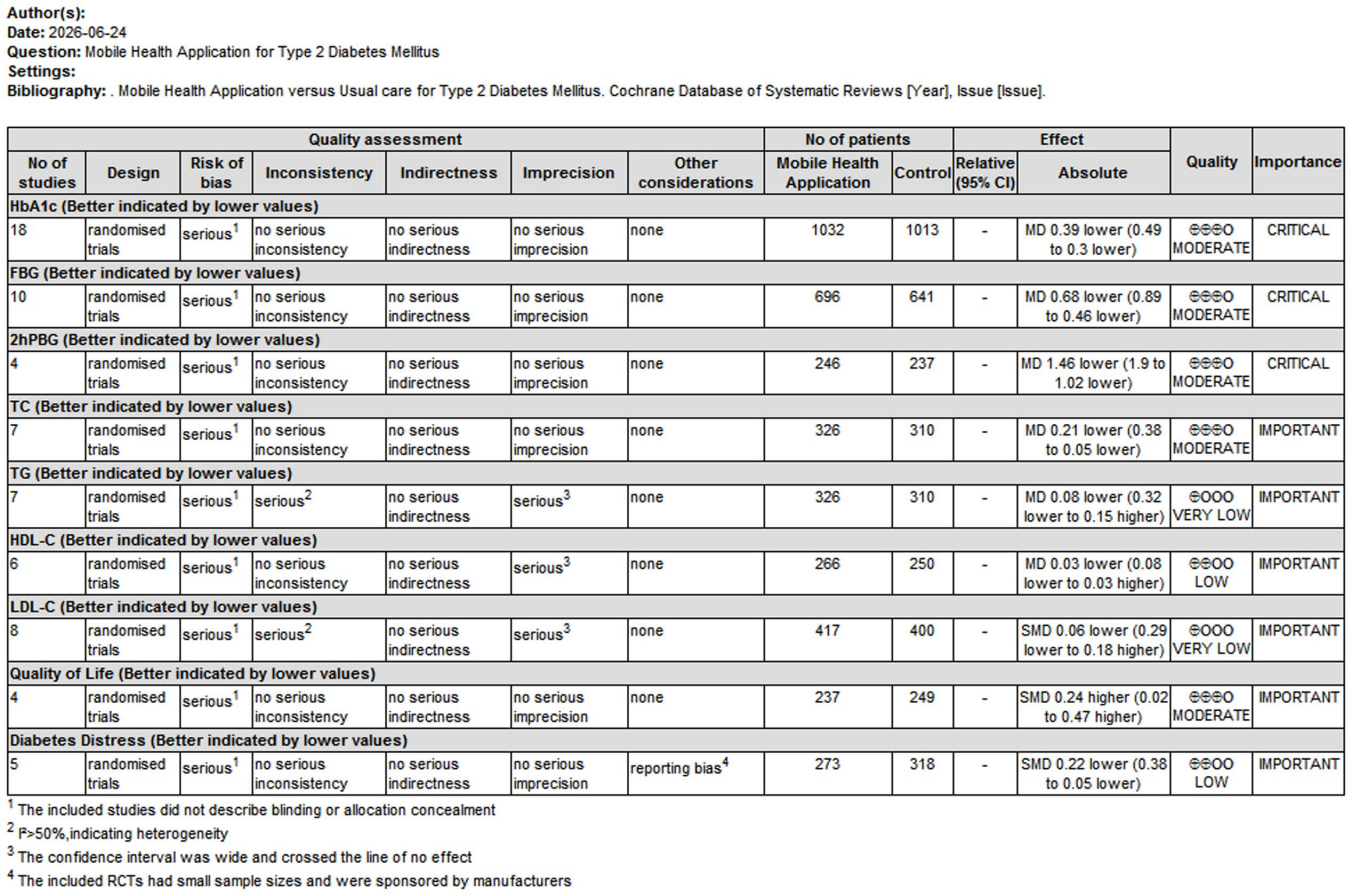
GRADE evidence grading chart.

An evaluation of the certainty of evidence for each outcome included in the meta-analysis revealed that all outcomes need further refinement in blinding methods. The GRADE assessment showed that the evidence for HbA1c, FBG, 2hPBG, TC, and quality of life was of moderate quality; the evidence for HDL-C and diabetes distress was of low quality; and the evidence for TG and LDL-C was of very low quality (Figure 13).

## 4 Discussion

### 4.1 Improvement in glucose metabolism

Our findings demonstrate that mHealth application interventions constitute an effective management strategy for T2DM, particularly in improving glycemic control. The effectiveness appears to be mediated by multiple mechanisms, including enhanced self-monitoring, personalized feedback, and improved self-efficacy. Glycemic control is crucial for delaying disease progression and preventing complications in T2DM. However, due to insufficient disease awareness, poor self-management behaviors, and limited access to traditional health education, glycemic control is often suboptimal (34). One study reported that the HbA1c target achievement rate (<7.0%) among medicated T2DM patients was only 49.20% (35). Our meta-analysis found that mHealth application interventions significantly improved glucose metabolism, with reductions in HbA1c and FBG levels, consistent with the findings of Wang et al. (16). The observed benefits of mHealth applications for glycemic control can be understood through established behavioral theories. According to self-determination theory (SDT) (36), mHealth applications may enhance intrinsic motivation for diabetes self-management by satisfying three basic psychological needs: Autonomy (through personalized goal setting and flexible self-monitoring), competence (through real-time feedback and skill-building modules), and relatedness (through peer support and provider communication features). Similarly, the health belief model (HBM) (37) suggests that mHealth applications improve self-management by increasing perceived susceptibility and severity (through visualized risk communication), enhancing perceived benefits (through positive reinforcement and outcome feedback), and reducing perceived barriers (through convenient access to education and support). These theoretical mechanisms are supported by our findings that applications with comprehensive features (including health recording, education, personalized guidance, and behavioral incentives) showed consistent benefits, whereas those with limited functionality yielded smaller effects. Future research could integrate digital twin technology and just-in-time adaptive interventions (JITAIs) into mHealth systems. Digital twins can continuously collect physiological, behavioral, and environmental data from early disease stages to construct individualized virtual health models, enabling real-time monitoring, risk prediction, and early warning (38). The integration of mobile health interventions with the risk warning capabilities of digital twins allows for the prediction of glycemic excursions, the remote supervision of patient-specific insulin doses and administration regimens, and the amelioration of insulin resistance (39). JITAIs can identify vulnerable states and opportunity windows in real time, delivering appropriately timed and tailored support to address challenges such as poor adherence (40). Relevant research has demonstrated that this intervention approach is effective in improving patients’ self-management behaviors and glucose metabolism (41). Combining these approaches could enhance personalized, precise, and long-term mHealth management for T2DM.

### 4.2 Unclear effects on lipid metabolism

T2DM patients often have dyslipidemia, which exacerbates insulin resistance, promotes atherosclerosis, and increases the risk of complications (42). Our meta-analysis showed that mHealth applications reduced TC but had no significant effects on TG, LDL-C, or HDL-C, consistent with some studies (18, 27, 29). Possible explanations include the fact that the majority of applications primarily focus on glucose monitoring, with insufficient attention to lipid-related factors, such as dietary fat intake, and a lack of targeted guidance. Moreover, improvements in lipid profiles usually require longer intervention periods and stricter dietary modifications, whereas the majority of included studies had durations of only 3–6 months (43). Differences in application functions, intervention intensity, baseline lipid levels, and sample characteristics may also contribute to heterogeneity. Future application designs should incorporate dietary recording and assessment modules, provide personalized feedback on lipid management, set lipid goals, and extend intervention durations to ≥12 months, with at least 6 months of follow-up, to clarify the effects on lipid metabolism.

### 4.3 Improvement in quality of life

Our meta-analysis found that mHealth applications improved quality of life in T2DM patients, which differs from some previous meta-analyses (8). This may be because the included applications offered features such as blood glucose monitoring, psychological support, and peer interaction, which directly alleviated anxiety and stress related to glycemic fluctuations, thereby improving daily functioning and subjective well-being. However, it is important to acknowledge the measurement variability across the four studies that reported quality of life, which used different instruments (EQ-5D, AQoL-8D, and the IQL test). Although the SMD approach appropriately standardizes these different scales and the low statistical heterogeneity (I^2^ = 31%) supports combining these studies, the clinical interpretation of SMDs may be less intuitive than MDs. The observed SMD of 0.24 represents a small but clinically meaningful effect (44), and the low heterogeneity suggests that, despite using different instruments, the studies showed consistent directions and magnitudes of effect. However, this meta-analysis included only four original studies, with a relatively limited total sample size, which may affect the robustness of the pooled effect estimates and the generalizability of the conclusions. Agarwal et al. (32) did not observe a significant improvement in quality of life, possibly due to the short intervention period and the application’s primary focus on glucose monitoring, without psychological or behavioral modules. In other studies, applications enabled patients to record daily data and visualize trends, with automatic alerts and feedback when parameters exceeded ranges, helping them adjust behaviors and stabilize blood glucose, thereby improving quality of life. Overall, preliminary evidence indicates positive effects, but the lack of standardized quality-of-life assessment limits direct comparability and clinical interpretability. Therefore, future high-quality RCTs should incorporate psychological support and adopt validated, standardized quality-of-life measures.

### 4.4 Reduction in diabetes distress

Diabetes distress is a specific psychological burden arising from the demanding self-management tasks required for diabetes, affecting up to 45% of patients and negatively impacting self-efficacy, self-management, treatment adherence, and glycemic control (45). However, it is often underrecognized in clinical practice. Our meta-analysis demonstrated that mHealth applications significantly reduce diabetes distress. This may be explained by the bidirectional relationship between diabetes distress and glycemic control; improved glycemic control can alleviate excessive worries about disease management (46). As novel self-management support tools, mHealth applications provide peer support, individualized guidance, and supervision, enhancing self-efficacy and confidence while reducing negative emotional responses, ultimately lowering diabetes distress. Yang et al. (47) showed that ecological momentary assessment (EMA) can capture dynamic fluctuations in diabetes distress and reveal real-time associations with perceived blood glucose, social interaction quality, physical symptoms, and sedentary behavior, with distress immediately leading to reduced self-management behaviors. These findings provide a rationale for developing intelligent, precision-based interventions for diabetes distress. Future research could combine EMA with JITAIs to build an adaptive intervention system that delivers timely, context-sensitive psychological support, cognitive restructuring, or mindfulness training when distress levels are elevated, transforming delayed, standardized support into immediate, on-demand, personalized care.

## 5 Limitations and future directions

First, the methodological quality of the included studies varied: Some studies inadequately reported random sequence generation and allocation concealment, and the majority of studies could not achieve blinding of participants, personnel, or outcome assessors due to the digital nature of the interventions, introducing potential performance and detection bias. Second, intervention durations were generally short, and long-term follow-up data were lacking to assess sustainability. Future studies should extend intervention durations and include follow-up periods of at least 6 to 12 months to assess the long-term maintenance of effects on glycemic and lipid control, quality of life, and the sustainability of behavioral changes. Third, the participants were predominantly middle-aged (45–61 years), limiting generalizability to younger or older adults. Future studies should broaden the age range of participants to encompass adolescents (such as those with type 1 diabetes) and older individuals (≥65 years of age). Fourth, although we did not perform formal subgroup analyses due to the limited number of studies in each category, several factors may influence the effectiveness of mHealth application interventions and warrant investigation in future research. These include the following: (1) age subgroups, as younger patients may have higher digital literacy and engagement; (2) intervention duration, as longer interventions may produce more sustained effects on lipid outcomes; and (3) application functionality profiles, as applications with integrated behavioral support and feedback may yield greater benefits than basic monitoring tools. Fifth, although the majority of studies showed positive effects on glycemic control, the number of high-quality, large-scale RCTs remains limited, and some studies had small sample sizes, reducing statistical power. Future well-designed RCTs are needed to confirm our findings.

## 6 Conclusion

This meta-analysis indicates that mHealth application interventions significantly improve glucose metabolism, quality of life, and diabetes distress in patients with T2DM, but their effects on lipid metabolism remain unclear. In this study, the meta-analyses of glycemic outcomes (HbA1c, FBG, and 2hPBG) included a relatively large number of studies, yielding stable pooled estimates with high credibility. Among the lipid metabolism indicators, only total cholesterol (TC) showed a positive result, but this was based on only seven included studies, suggesting weak evidence. Although quality-of-life outcomes also demonstrated positive effects, they were derived from merely four studies with heterogeneous measurement tools, and the robustness of the pooled effect was therefore insufficient. Some data were derived from available statistics, which may have introduced minor deviations from the original data. Future efforts should focus on integrating behavioral management, psychological support, social interaction, and real-time feedback modules, and incorporating digital twin technology and JITAIs into mHealth application design and practice to advance intelligent, personalized, and proactive diabetes care. User-friendly, high-performance applications tailored to patients’ age, disease duration, and cultural background are needed. Moreover, large-scale, multicenter, high-quality RCTs are required to further elucidate the effects of mHealth applications on glycolipid metabolism and quality of life, thereby providing more robust scientific evidence.

## Data Availability

The original contributions presented in this study are included in the article/supplementary material, further inquiries can be directed to the corresponding author.
